# Why critical psychology and the neurodiversity movement need each other

**DOI:** 10.3389/fpsyg.2024.1149743

**Published:** 2024-01-18

**Authors:** Eleanor Thomas

**Affiliations:** Prescott College, Critical Psychology and Human Services, Prescott, AZ, United States

**Keywords:** autism, Autistic, critical psychology, neurodivergent, neurodiversity

## Abstract

Critical psychology is a discipline that can be defined in a variety of ways, though common themes include critiquing mainstream psychology as well as critiquing society at large and engaging in social change to respond to those critiques. The neurodiversity movement is a political movement that emerged in response to the dehumanizing treatment of Autistic and other neurodivergent individuals by society in general and by mainstream psychology specifically. In this article, I describe five ways in which critical psychology and the neurodiversity movement can and have begun to benefit from each other: (a) by critical psychologists embracing neurodivergent epistemologies in the way they embrace other diverse epistemologies; (b) by resisting attempts within mainstream psychology to pathologize difference or “treat” these so-called psychopathologies by modifying behavior; (c) by practitioners developing cultural competency around neurodivergent culture within the psychotherapeutic practice; (d) by challenging the homogenization and whitewashing within the neurodiversity movement through leveraging lessons from within various critical psychologies; and (e) by researchers and practitioners recognizing and combatting instances of ableism embedded in other forms of oppression and within the field of critical psychology itself.

## 1 Introduction

Critical psychology as a discipline has many definitions with common themes: the critique of mainstream psychology, the critique of society at large, and the work required to bring about social change in response to those critiques (Hook, 2005). Various related fields address specific concerns of populations most impacted by the failures of society and mainstream psychology, including community psychology, liberation psychology, and others. “Mainstream psychology,” it must be noted, does not refer to a monolith of thought within the academic and professional field of psychology (as no such monolith exists). Rather, it refers to the amorphous collection of pervasive beliefs and assumptions common amongst academic psychologists and clinical practitioners that necessarily shift over time. These very beliefs and assumptions influence what is considered to be “controversial” at any given point in time, and what presents as a “bold claim” through this mainstream lens may be a matter-of-fact observation from the lived experience of a minoritized perspective. The neurodiversity movement is a political movement Autistic^1^ advocates started in response to dehumanizing treatment by society and mainstream psychology (Walker, 2021). The neurodiversity movement has since grown and is an inclusive movement, encompassing the full range of neurological variation among humans. The Autistic population specifically has been let down by mainstream psychology in ways that critical psychology speaks to, and given my positionality as an Autistic individual, this population will therefore be my focus.

Hook (2005) proposed engaging in critical psychology from a “psychopolitical” perspective. He argued that the psychological and the political are inherently related and cannot be separated. I similarly argue that the field of critical psychology and the neurodiversity movement are interconnected. I do not view the Autistic experience through the purely psychological (and psychopathological) lens of mainstream psychology but rather as an inherently “political” experience, the same way that other marginalized groups have had their psychological experiences politicized. Others have similarly argued that autism is an inherently political identity as a result of emergent Autistic culture and community (Chapman, 2016).

Neurodiversity is a relatively new topic of scholarship, and further research is needed in areas that could lead to actionable recommendations, including the topic of handling neurodiversity in the workplace (Doyle and McDowall, 2022). The term “neurodiversity” and the neurodiversity paradigm have started to gain traction within the field of psychology, but there is still widespread misunderstanding and misuse of the terms (Dwyer, 2022). The term *neurodiversity* refers to the diversity of human minds, a biological reality rather than a philosophical perspective or political movement (Walker, 2021). The *neurodiversity paradigm* refers to a perspective on neurodiversity that this diversity is natural, valuable, and subject to the same social dynamics—and oppression—as other forms of human diversity. The *neurodiversity movement* is a political movement. Recent research indicates that more attention to the neurodiversity paradigm is warranted (Pellicano and den Houting, 2022).

Although a complete treatment of opportunities for collaboration between critical psychology and the neurodiversity movement is beyond the scope of this article, I will present five ways that the two can and have already begun to benefit from each other: (a) by critical psychologists embracing neurodivergent epistemologies the way they embrace other diverse epistemologies; (b) by resisting attempts within mainstream psychology to pathologize difference or “treat” these so-called psychopathologies; (c) by practitioners developing cultural competency around neurodivergent culture within the psychotherapeutic practice; (d) by advocates leveraging lessons from critical psychologies to challenge homogenization and whitewashing in the neurodiversity movement; and (e) by recognizing and combatting ableism embedded in other forms of oppression and within critical psychology scholarship.

## 2 Embracing neurodivergent epistemologies

Critical psychologists have criticized the epistemological gatekeeping of mainstream psychology, wherein the established field embraces knowledge systems that are fundamentally at odds with the lived experience of those marginalized due to their race, gender, or other aspects of identity (Bohan, 2002; Paredes-Canilao et al., 2015; Stevens, 2015). Consequently, subdisciplines of critical psychology have emerged in specific contexts and in response to injustices felt by these different groups (Teo, 2015). Neurodivergent individuals specifically, by virtue of having “a mind that functions in ways which diverge significantly from the dominant societal standards of ‘normal”’ (Walker, 2021, p. 38), also have divergent ways of knowing, or epistemologies. The term “neurodivergent” was coined by activist Kassiane Asasumasu in 2000 and is intentionally inclusive of any neurological difference. The term “neurotypical” refers to anyone whose neurocognitive functioning falls within dominant societal standards. Naturally, the line between the two is subjective.

Monique Botha (2021) described their experience of gatekeeping and dehumanization when trying to enter the field of psychology as an Autistic person. For Botha, an Autistic autism researcher, it is not enough to simply “invite” Autistic participants to engage in research. Psychology must first reckon with the violent, objectifying, and dehumanizing accounts of autism that are standard in psychological education. Further, Botha described having their own “objectivity” and credibility challenged due to their positionality as an Autistic person. A critical psychologist might argue that this positionality renders Botha *especially* qualified to study these topics. This questioning of a researcher’s objectivity based on positionality occurs across the field of psychology. Even so-called “progressive” fields like Western feminism engage in a form of epistemic violence against Majority-World women by undermining the validity of their perspectives, driven by paternalism and ignorance (Kurtiş and Adams, 2017). The commonness of this epistemic violence and the lack of ill intent of mainstream psychologists perpetuating it does not lessen its harmful impact.

Ensuring that Autistic researchers are involved and respected in the field of autism research is one step toward embracing neurodivergent epistemologies. The research methodology employed to study autism and Autistic people also influences the extent to which these perspectives are included. Botha (2021) touched briefly on participatory and action research (PAR) in the context of autism research, which is another way to embrace neurodivergent epistemologies—by directly involving Autistics in the research process itself, especially in ways that generate direct benefit for the Autistic community. PAR is “an approach to [i]nquiry which … involves researchers and participants working together to understand a problematic situation and change it for the better” (Participatory Methods, n.d., para. 1). This approach has proven successful in response to humanitarian crises faced by Central American refugees, Black women in South Africa, and those impacted by occupation in Israel/Palestine (Lykes, 2013; Segalo et al., 2015). A key result of each of these research projects was that participants (or *co-researchers*) came away with something of direct benefit to themselves or their local community, namely maps, embroideries, and other tangible collective projects of value to the specific communities, rather than the researchers simply “extracting” knowledge for export to academia.

The Participatory Autism Research Collective (PARC) promotes participatory action research projects within autism research. PARC aims to build a community network for those who wish to see more involvement of Autistic people in autism research (The Participatory Autism Research Collective, n.d.). Neurodivergent researchers have also proposed “neurodivergent storying” approaches to create a more welcoming space for neurodivergent individuals (Rosqvist et al., 2023). A common refrain in the disability and Autistic communities is the phrase, “nothing about us without us.” By this logic, it could be argued that *all* autism research should be conducted by, and for, Autistic people. Any step in that direction would be an improvement over the *status quo*.

Finally, Autistic and neurodivergent individuals are not the only potential beneficiaries of their epistemological inclusion. As Arnett (2008) argued, the overrepresentation of White people from the United States among the psychologists cited and subjects studied in U.S. psychology harms not only those left out of the conversation but also those centered in it. Arnett gave examples of how the variety in educational systems, family structures, etc. worldwide is a valuable source of wisdom for anyone, including those from historically dominant groups. I argue that neurodivergent wisdom can similarly benefit the dominant population. Neurodivergent people face unique challenges and have developed creative resiliencies as a result, such as Autistic people disproportionately bearing the brunt of compensating for communication style differences (Milton, 2012).

## 3 Resisting attempts to pathologize and “normalize” difference

Another area of mutual benefit is through resisting the pathologization of difference and attempts to eliminate behavioral differences through “normalization.” Best and Kellner defined normalization in this context as “the elimination of all social and psychological irregularities and the production of useful and docile subjects through a refashioning of minds and bodies” (as cited in Hook, 2004, p. 227). Critical psychology names and confronts harmful attempts at normalization in other contexts already, including heteronormativity. Heteronormativity is “the cultural/political privilege of heterosexuality as the axis of intimacy and the pillar of social participation and organization” (Peñaloza and Ubach, 2015, p. 341). Psychologists have historically enforced this privilege, including through so-called *conversion therapy*, whereby psychologists have attempted to “cure” those who did not conform. Homosexuality was not removed from the Diagnostic and Statistical Manual (DSM) until 1973 (Drescher, 2015). Cisgenderism and the pathologization of uncommon gender expressions in children remain prevalent in psychological literature (Ansara and Hegarty, 2012).

Neurodivergent individuals face similar pressure to conform to neuronormative behavioral ideals (Walker, 2021). One of the “treatments” offered to Autistic children is applied behavior analysis (ABA), a systematic approach to modifying the behavior of Autistic children by coercing or forcing them to behave more “normally.” The similarities to conversion therapy for homosexual children are not just coincidental; the creator of ABA, Ole Ivar Lovaas, was also involved in the Feminine Boy Project in the 1970s, and many of his behavioral “interventions” have been used in both contexts (Gibson and Douglas, 2018).

The criticism of ABA within the Autistic community is well-founded. Research links experiences in ABA to increased symptoms of post-traumatic stress disorder in Autistic adults (Kupferstein, 2018). Furthermore, at the time of writing, there is still one facility in the United States (the Judge Rotenberg Center in Massachusetts) that employs electric shock on Autistic people as punishment to enforce neuronormative behavior, despite an initial FDA ban in 2020 (Young and McMahon, 2021). Given that these residents are among the most vulnerable members of the Autistic population, those who are non-speaking or with high support needs (overlapping but not identical groups), the lack of public will to end this abuse is a significant political and human rights issue. These most vulnerable groups are also those most likely to endure abuse in ABA programs well into adulthood (Shkedy et al., 2021).

Even when ABA programs do not last beyond childhood, the harm that they cause may last a lifetime as Autistic people internalize the message that their behavior is wrong. Hook (2004) invoked Michel Foucault’s notion of “disciplinary power” to describe how institutions of power, including psychology itself, function. A defining characteristic of disciplinary power is the process of self-surveillance and self-discipline whereby the enforcement of norms becomes internalized. Autistic people do report “masking” or intentionally suppressing aspects of themselves to conform, to the detriment of their mental health (Pearson and Rose, 2023). This is also how ABA and other efforts to repress Autistic behavior can be internalized to the detriment of the individual. Queer theorists speak of “disarticulating,” abolishing, or disrupting the logic underpinning the concept of normative sexuality, not just displacing the line between what is considered normal and abnormal (Peñaloza and Ubach, 2015). Such a disarticulation—rather than displacement—is likewise called for when it comes to harmless neurodevelopmentally based behavioral differences.

## 4 Developing neurodivergent cultural competency within therapeutic practice

Critical psychologists (and increasingly mainstream psychologists) have recognized the importance of developing cultural competence within psychotherapeutic practice. Various marginalized groups, including Filipino Americans marginalized by a history of colonization in the Philippines and internalized colonialist ideals, have discussed the importance of having therapists informed about and sensitive to their circumstances (David and Okazaki, 2006). Autistic individuals have similarly requested that non-Autistic therapists develop humility toward and learn from Autistic individuals (Bulluss, 2021; Walker, 2021).

Culturally competent psychotherapists must also be familiar with the minority stress model. This model, though not developed with Autistic people in mind, has been shown to account for a large proportion of distress experienced by Autistic individuals (Botha and Frost, 2020). The minority stress model predicts that individuals within a marginalized group will experience heightened stress resulting in poorer mental health outcomes simply by virtue of the microaggressions and daily barriers associated with minoritized status. This research shows that by taking this model into account, a large proportion of the distress commonly attributed to autism as a “disorder” could be attributed to social factors associated with minoritized status and the effects of marginalization.

Numerous outdated and harmful autism theories linger in therapeutic practice. Research has challenged claims that Autistic people lack empathy or communication skills. Rather, having a different neurocognitive style from the majority results in challenges communicating with and empathizing with *the majority*, but not necessarily with other Autistic people (Milton, 2012; Crompton et al., 2020). Claims that Autistic individuals lack a “theory of mind” have also been discredited, yet some practitioners still repeat these harmful stereotypes (Gernsbacher and Yergeau, 2019).

## 5 Challenging homogenization and whitewashing within the neurodiversity movement

The neurodiversity movement can also benefit from the broader perspective of critical psychology. Autistic advocates have been criticized for insufficient intersectionality in their critiques of mainstream psychology and society, especially along race and gender lines (Giwa-Onaiwu, 2020). Autistic people have been stereotyped as White, cisgender, heterosexual males, supposedly supported by the available prevalence data (Centers for Disease Control and Prevention, 2021). Critics have argued that gendered differences in autism diagnosis rates may reflect unequal access to diagnosis for Autistic people of marginalized genders and a lack of education on the part of diagnosticians rather than an inherent difference in prevalence. Some psychologists and those in the Autistic community have developed checklists and descriptions for “female autism” compared with the more stereotypical “male autism” (Sarris, 2022). The concept of “male” and “female” autism problematically reinforces the gender binary in a population that has significant overlap with non-binary and transgender populations (Warrier et al., 2020). This is another situation that calls for “disarticulation” rather than “displacement” – in this case, of the boundary between “male” and “female” Autistic traits in diagnostic tools/processes and public discourse (Peñaloza and Ubach, 2015).

The neurodiversity movement itself risks homogenization and consequent erasure of the experiences of those who are minoritized within the Autistic community, especially Black and Indigenous Autistic people. Such a risk has been considered within other social movements, including Black psychology (Stevens, 2015). As Stevens noted, homogenization may be a necessary oversimplification early on in a social movement’s formation to gain traction. Nevertheless, any social justice movement will ultimately fail if it cannot meet the needs of its most marginalized members.

The neurodiversity movement does exhibit harmful effects of homogenization and “whitewashing”—that is, centering the White Autistic experience. One example is the backlash against ABA. While ABA is widely recognized as harmful within the Autistic community, the conversation is more nuanced for Autistic people facing other societal barriers. For instance, some parents of Black Autistic boys have a more complicated relationship with behavior-based programs, since their children have historically experienced police brutality at much higher rates, and “behavior” can be a matter of life and death for them (Aucademy, 2021; Hammond, 2022; Hutson et al., 2022). Further, racial disparities exist within autism research, leading to poorer health outcomes for Black Autistic people especially (Jones and Mandell, 2020). More robust collaboration between the neurodiversity movement and critical psychologists could lead to a broader understanding of intersectionality and its implications by Autistic advocates, a trend that is already starting (Rowan Center for Neurodiversity, 2022).

## 6 Recognizing and combating ableism within critical psychology

In this final section, I challenge ableist trends in critical psychology and suggest that ableism – that is, the systemic marginalization and oppression of disabled individuals – is at the root of other forms of oppression that critical psychology aims to combat, therefore warranting especially close consideration.

In the same way that neurodiversity movement advocates are not immune to engaging in racism, sexism, or other forms of bigotry, critical psychologists may end up reinforcing harmful ableist tropes in their work. For example, some critical researchers casually invoke bipolar disorder and narcissistic personality disorder, using them as metaphors for a sick society (Mentinis in McDonald et al., 2007; Dafermos et al., 2013). While this strategy may get the point across to a presumed neurotypical audience, it alienates neurodivergent readers, especially those carrying these diagnoses. That these authors assume that their readers will recognize these conditions as metaphors for sickness and dysfunction, implicitly assuming that their readers will not *themselves* be members of these “disordered” populations, reflects precisely the sort of casual ignorance and ableism that Botha (2021) described as a barrier to entry into the field of psychology for Autistic people. Thus, the lack of neurodivergent individuals and awareness of the neurodiversity movement in psychology, even critical psychology, becomes self-reinforcing. This is especially evident in anti-psychiatry movement within critical psychology, which has been criticized for erasing real disability and leaving individuals vulnerable. The neurodiversity paradigm does not warrant the erasure of categories like autism, but the end to pathologization of them (Chapman, 2020).

Finally, I argue that all forms of bigotry and oppression rest on assumptions of superiority and inferiority of various groups of people (Baggs, 2016). As a result, liberation for those who are considered “disordered” can have a broadly emancipatory impact. Once again, a form of disarticulation, not just displacement, is required when it comes to neurodevelopmental and cognitive differences currently classed as disorders. Such a disarticulation would also dismantle the underpinnings of other forms of cultural chauvinism, benefitting all who seek a more socially just world.

## 7 Discussion

This exploration of the potential for mutual benefit between critical psychology and the neurodiversity movement began with the assertion that the psychological and the political are inherently connected (Hook, 2005). Critical psychologists have historically considered culturally divergent epistemologies, and I proposed that critical psychologists embrace neurodivergent epistemologies following this same model. Concretely implementing this suggestion involves actively including Autistic voices in research and educational institutions to stretch the boundaries of what is considered “normal” or acceptable for communication style and knowledge production methodology.

Psychology has historically been relatively inaccessible to Autistic would-be researchers who could benefit the field of autism research with their lived experience. This expertise through lived experience from Autistic researchers is essential to resist attempts to erase and pathologize neurodivergent experience, especially Autistic experience. Additionally, the onus is on all psychology professionals to openly question the use of so-called therapies that seek to minimize behavioral differences not impacting quality of life. An increased presence of Autistic psychologists in research and academia would also support the effort to increase therapist cultural competence with neurodivergent individuals through the teaching and training of new psychologists. Concretely, training programs for new clinicians must teach and model cultural humility around neurodivergent culture, and continuing education on this topic should be offered for current clinical professionals.

Further, increased engagement by critical psychologists in the neurodiversity movement would potentially bring diverse perspectives to a movement that is at risk of homogenization, especially given the emphasis on intersectionality within certain subdisciplines of critical psychology. To that end, I urge researchers and professionals to actively include Autistic voices who are also Black, Indigenous, and from gender minorities for a fuller and more accurate reflection of the Autistic population. And finally, professionals must actively challenge the use of metaphors and habits of thought in academic psychology, including critical psychology, that reinforce ableist tropes. With the understanding that the psychological and the political can never truly be separable, I have argued that the neurodiversity movement *as* a sociopolitical movement belongs squarely in the awareness and work of critical psychology.

## Data availability statement

The original contributions presented in this study are included in this article/supplementary material, further inquiries can be directed to the corresponding author.

## Author contributions

The author confirms being the sole contributor of this work and has approved it for publication.

## Footnotes

1 The capitalization of Autistic here and throughout is intentional and signifies respect, analogous to the capitalization of other identity groups.
